# Impact of COVID-19 pandemic quarantine on dietary behaviors and lifestyle of Saudi adults in Jeddah, Kingdom of Saudi Arabia

**DOI:** 10.15537/smj.2022.43.8.20220217

**Published:** 2022-08

**Authors:** Eilaf H. Braiji, Eman A. Abduljawad, Amani A. Alrasheedi

**Affiliations:** *From the Food and Nutrition Department (Braiji, Abduljawad, Alrasheedi), Faculty of Human Sciences and Design, King Abdulaziz University, Jeddah; and form the Department of Food and Nutrition Department (Braiji), Taibah University, Al Madina Al Munawara, Kingdom of Saudi Arabia.*

**Keywords:** COVID-19, quarantine, dietary behaviors, lifestyle

## Abstract

**Objectives:**

To investigate how the coronavirus disease 2019 (COVID-19) pandemic quarantine affected Saudi adults’ dietary behaviors and lifestyles in Jeddah, Saudi Arabia.

**Methods:**

A retrospective cross-sectional study using an electronic questionnaire was adopted. A total of 476 Saudis residing in Jeddah were involved with the investigation. The study was carried out from March to May 2021. The questionnaire addressed dietary behaviors (types of foods consumed) and lifestyle behaviors (food shopping, physical activity, and sleep quality).

**Results:**

The study participants aged 18-65 years, 70.2% were females, mainly non-infected with coronavirus previously (88.7%). Fast food consumption as (pizza and burgers) decreased significantly during the quarantine period. However, consumption of snacks, sugars, and pastries increased significantly during the quarantine period. This could be attributed to the boredom and distress experience during this period. The results showed that participants reported a significant decreases in daily physical activity and shopping during the quarantine period. There was also a significant change in their sleep quality. Moreover, there were significant differences between males and females concerning eating snacks, healthy foods, sweetened beverages, pastries, physical activity, daily activities (housework and shopping), and smoking behavior.

**Conclusion:**

The COVID-19 quarantine period significantly affected Saudi adults’ healthy eating behaviors and lifestyle.

**C**oronavirus disease 2019 (COVID-19) is caused by severe acute respiratory syndrome coronavirus 2 virus (SARS-CoV-2). In December 2019, an outbreak of SARS-CoV-2 infection was discovered in Wuhan, China, and has spread across the globe.^1^

In view of the surging number of COVID-19 patients in China and globally, the outbreak was deemed a world health emergency by the Emergency Committee of the World Health Organization (WHO) in January 2020. To contain the increase prevalence of the virus, governments around the world declared national emergencies and imposed stringent measures of containment. The quarantine period was extended for several months in some countries.^2^

The increasing rates of new positive cases of COVID-19 in many countries and the long periods of quarantine have led to the establishment of stay-at-home laws. The impact of self-isolation and physical distancing on the lives of people worldwide affected individuals’ eating habits and social and psychological behaviors.^3^

Saudi society is characterized by a great interest in travel, social gatherings, and family relationships, especially on holidays and social occasions. After lockdowns were instated owing to COVID-19 with suspension of travel, there have been limited opportunities to leave the house. This has in turn affected mental health and has resulted in many changes to the lifestyles of Saudi families as concluded in a study conducted by Al-Saleh et al.^4^ Two main changes can occur when people are isolated and remain at home, and are unable to lead a normal life. The first lifestyle change involves reducing physical activity due to restrictions on moving outside homes. Homestays can lead to major lifestyle changes that may adversely affect health, including the adoption of sedentary lifestyle, unhealthy sleep, and smoking behaviors. Another change involves eating behaviors, such as food hoarding or developing eating disorders. During lockdowns, there has been limited access to fresh dietary sources and a decrease in the variety of available food groups. Alternatively, individuals may turn to eating preserved food products exhibiting high calories with low nutritional values. Boredom caused by reduced or lost work and media reports on COVID-19 also make life stressful. Boredom or stress can result in cravings, binge eating, and increased caloric intake, ultimately negatively affecting overall health.

Researchers believe that limited access to daily activities such as shopping or walking may lead to reduced in-take of basic foods, especially foods such as vegetables, fruits, and fish. These foods are being replaced by highly processed items, including fast foods, prepared foods, and ready-to-eat foods, cereals, and snacks, which are usually rich in sugars, fats, and salt.^5,6^

As many have been aware up to this point, COVID-19 is the modern disease that spreads from one individual to another. It causes a serious public health problem worldwide with its mild to severe respiratory effects, especially on the elderly. As a result of the pandemic, the changes in the lifestyle and behaviors were addressed in the present paper. With this information now available, the importance of the paper lies in spreading awareness and improving public knowledge of viral diseases and their impact, not only on the human body but also on the behavior and lifestyle of individuals. Therefore, basic knowledge and understanding of any changes in habits, changes in social behaviors of community members, changes in shopping patterns and food consumption constitutes a real reason to raise awareness levels among citizens in Saudi Arabia in general, and the city of Jeddah in particular. In the current study, we aimed to ascertain the impact of quarantine owing to the COVID-19 pandemic on food habits, behaviors, food shopping, and consumption patterns, as well as changes in the lifestyle habits of Saudi adults living in Jeddah.

## Methods

A descriptive cross-sectional study was carried out among 476 Saudi male and female adults. The inclusion criteria were Saudi male and female living in the city of Jeddah, aged between 20 to 60 years, and who could shop and prepare food without assistance. The exclusion criteria were non-Saudis, age under 20 years or over 60 years, individuals not living in Jeddah, pregnant and breastfeeding women, people who need help preparing and shopping for their food, and individuals with chronic diseases. The study protocol was approved by the Research Ethics Committee of the Biomedical Ethics Unit at King Abdulaziz University (KAU), Jeddah, Saudi Arabia (Reference No. 140-21).

The sample was calculated based on the latest statistical data provided by the General Authority for Statistics in 2010 for the population of the Kingdom of Saudi Arabia, categorized by cities. The total number of Saudi men and women in Jeddah is 1,716,574 people.^7^ The sample size was computed utilizing the following formula: n=(z2pq)/(d2). Assuming the proportion of the population given the maximum possible sample size required (*p*=0.50), with a confidence level of 95% and a margin of error of 5%, the sample size was computed using an electronic calculator.^8^ The total sample size was 385 individuals; and the overall number of participants was 826. The data were filtered in accordance to the inclusion and exclusion criteria; the total final sample was 476 participants.

The questionnaire in this study has been published and validated by Chopra et al,^9^ and translated into Arabic language. Google forms were used to build the survey. The questionnaire was disseminated via social media (WhatsApp, Twitter, KAU university e-mail) from March to May 2021. On the first page, the voluntary nature of participation with informed consent was explained alongside the rationalization of the purpose and objectives of the project. Subsequently, respondents could continue to complete the questionnaire.

The utilized survey included a total of 44 questions, which were categorized into 3 sections, as follows: i) Sociodemographic and health data: This section included 15 questions that addressed age, gender, location of residence, nationality, educational status, monthly family stipend, marital status, health status, weight, and height. ii) Food habits and behaviors: This section included 23 questions regarding participants’ frequency of food consumption and eating habits during and after COVID-19 quarantine as well as information regarding lifestyle habits (grocery shopping, physical activity, quality of sleep, time spent daily using social media and electronic devices, and level of anxiety). iii) Other lifestyles and practices: This section included 6 questions addressing the reasons for lifestyle habits alteration throughout COVID-19 quarantine, with the option to choose more than one answer for each question. iv) Anthropometric measurements: Self-reported data on height and weight (last known prior to the epidemic) were utilized to compute and interpret body mass index (BMI) using the Quetelet formula: body mass (kg)/height (m^2^) and interpreted in accordance to WHO criteria. Four categories were identified: underweight (BMI <18.5 kg/m^2^), normal weight (BMI 18.5 to <25.0 kg/m^2^), overweight (BMI 25.0 to <30 kg/m^2^), and obese (BMI 30 kg/m^2^).

To ascertain the reliability of the study scale Cronbach’s alpha (α) test was used as it can help in determining whether all items are measuring the same factor and to measure the internal consistency of a survey instrument.^11,12^ The Cronbach’s α acceptable scores ranged between 0.70 and 0.95, although, low scores could be attributed to insufficient questions, poor levels of inter-relation between items or constructs heterogeneity.^13^ Hence, in this study, the scores of the Cronbach’s α test; Overall reliability score for all items (5-Point Likert scale) was obtained 0.700 and 0.702 for the questions of during and after the COVID-19 quarantine respectively, which is suggestive of a good internal consistency.^14^ According to Nunnally,^15^ a value of α that is equivalent to or over 0.70 is indicative of the reliability of the scale’s items.

The inter-construct correlation validity for the questionnaire (during-after: 23 items); from which we found that all correlation coefficient was significant with *p*<0.01, ranged between 0.505 and 0.878.

### Statistical analysis

The statistical analyses were carried out using the Statistical Package for the Social Sciences, version 26.0 (IBM Corp., Armonk, NY, USA). Descriptive statistics (such as, frequency, percentage, mean, and standard deviation) were used to present data, and the Chi-square test and correlation coefficient were computed to assess the association between the study variables. A *p*-value of <0.05 was deemed statistically significant.

## Results

In this study, the overall sample size was 476 participants, all of whom were Saudis living in Jeddah (70.2% women and 29.8% men). The age group 18-25 years made up 38.2% of the total sample and the age group 56-65 accounted for 3.2%. As for education levels, 20.4% had a secondary school diploma or less, 58% had an undergraduate degree, and 21.6% had a postgraduate degree. Regarding income, 56.3% reported earning 5,000 to <10,000 riyals. In total, 48.9% of participants were unmarried and 45.4% were married (Table 1).

**Table 1:** - Sociodemographic and health data of study participants (N=476).

Variables	n	%
***Gender***		
Male	142	29.8
Female	334	70.2
***Age (years)***		
18-25	182	38.2
26 - 35	121	25.4
36 - 45	94	19.7
46 - 55	64	13.4
56 - 65	15	3.2
***Educational level***		
High school or less	97	20.4
Bachelor’s degree	276	58.0
Master’s or Ph.D.	103	21.6
***Monthly family income***		
Less than 5000 riyals	55	11.6
From 5,000 to <10,000 Saudi Riyals	153	32.1
>10,000 Saudi Riyals	268	56.3
***Marital status***		
Single	233	48.9
Married	216	45.4
Divorced or widowed	27	5.7
***Do you need help with shopping or preparing and preparing food?***		
Yes	207	43.5
No	269	56.5
***Have you had the corona virus?***		
Yes	54	11.3
No	422	88.7
***During the quarantine period due to the Corona pandemic, has there been a change in your weight?***		
No change in weight	46	9.7
Lost weight	136	28.6
I gained weight	85	17.9
I do not know	209	43.9
Weight, kg (self-reported)	69.36±19.030	
Height, cm (self-reported)	162.55±9.129	
Body mass index (BMI)	26.10±6.181	
***BMI (kg/m^2^)***		
Underweight	35	7.4
Normal	232	48.7
Overweight	113	23.7
Obese	96	20.2

The association between eating habits and lifestyle behaviors during and after COVID-19 quarantine were studied. Results show that the intake of unhealthy foods raised significantly in women compared to men during and after quarantine. Unhealthy lifestyle habits included intake of fast food, French fries, snacks, sugar-sweetened beverages, and pastries. Moreover, healthy lifestyle behaviors of consuming healthy foods during quarantine increased significantly in women and consumption of milk and dairy products increased in women after quarantine.

Physical activity was significantly increased in women during the quarantine, such as doing 30 minutes of exercise per week or participating in household chores. Women showed a significant increase in working hours after the quarantine period. Women also showed a significant increase in sleep duration after quarantine.

Females had substantially higher rates of stress and anxiety after the quarantine period. A significantly higher rate of women reported smoking during and after quarantine (Table 2).

**Table 2:** - Study participants dietary habits and behaviors, food shopping and consumption pattern, and lifestyle behaviors during and after the COVID-19 quarantine (N=476).

Behavior	Time	Mean	Std. deviation	T-Test	*P*-value
How often do you commit to eating 3 main meals and 2 snacks?	During	2.07	1.464	-0.716	0.475
	After	2.11	1.442		
How often do you eat fast food such as pizza, burgers as daily main meals?	During	2.06	0.997	-3.497	0.001^**^
	After	2.21	0.891		
How often do you eat french fries?	During	2.04	0.893	-0.561	0.575
	After	2.06	0.862		
How often do you eat snacks (such as popcorn, chips, and so on)	During	2.43	1.183	5.444	0.000^**^
	After	2.18	1.080		
How often do you eat fruits and vegetables?	During	2.84	1.216	1.061	0.289
	After	2.80	1.234		
How often do you eat healthy foods (eggs, whole wheat, nuts, fruits and vegetables) in your daily meals?	During	2.84	1.374	-1.147	0.252
	After	2.88	1.386		
How often do you have 2-3 servings of milk or its products (such as laban, yoghurt, and cheese)	During	2.93	1.319	0.507	0.613
	After	2.92	1.318		
How often do you eat one or more servings of units of eggs or meat per day	During	3.14	1.186	0.258	0.797
	After	3.13	1.203		
How many spoons of sugar do you add to foods and drinks per day?	During	2.06	0.930	3.913	0.000^**^
	After	1.95	0.888		
How often do you soft or sweetened beverages (juices, sodas, energy drinks, so on)?	During	2.31	1.291	1.41	0.159
	After	2.26	1.258		
How often do you eat pastries (cakes, donuts, bites, pastries, sweets, chocolate, and so on)?	During	2.68	1.211	4.794	0.000^**^
	After	2.47	1.080		
How often do you eat fast food because of boredom, distress and the desire to eat without hunger?	During	2.13	1.160	2.997	0.003^**^
	After	1.99	1.016		
How often do you do 30 minutes of physical activity per week (such as walking, cycling, dancing, so on)?	During	2.20	1.288	-2.514	0.012^*^
	After	2.34	1.282		
How often do you contribute in (cooking, laundry, or cleaning) at home?	During	3.12	1.527	2.691	0.007^**^
	After	3.01	1.562		
How often do you contribute to daily activities such as (shopping grocery, neighborhood walk?	During	2.21	1.209	-5.364	0.000^**^
	After	2.45	1.177		
How many hours is your daily time sitting at work?	During	2.90	1.756	-5.505	0.000^**^
	After	3.21	1.752		
How many times do you break while working during office hours (rest time 10 minutes)?	During	2.50	1.310	-1.52	0.129
	After	2.56	1.224		
How many hours do you spend daily watching television or mobile phone for social media or playing video games?	During	3.28	0.918	8.261	0.000^**^
	After	3.01	0.962		
How many hours do you sleep daily?	During	2.03	0.681	5.709	0.000^**^
	After	1.86	0.642		
Is there any change in your quality of sleep?	During	2.46	1.080	-5.805	0.000^**^
	After	2.74	1.088		
How many times do you feel stress or anxiety in a day?	During	2.74	1.116	-0.424	0.671
	After	2.76	1.106		
Do you smoke cigarettes, shisha or almuasal or electronic cigarettes?	During	1.23	0.667	-0.143	0.887
	After	1.23	0.665		
Do you received any help to maintain a healthy lifestyle from family and friends?	During	3.05	1.353	1.261	0.208
	After	3.01	1.342		

^**^ Significant at 0.01, ^*^Significant at 0.05, NS: not significant (*p*>0.05), Std: standard

Nevertheless, the association between eating habits and lifestyle behaviors throughout and after quarantine according to gender was studied(Table 3).

**Table 3:** - Association between the dietary habits and behaviors, food shopping and consumption pattern, and lifestyle behaviors during and after the COVID-19 quarantine and gender (N=476).

Behaviors	During		After	
	Chi-square	*P*-value	Chi-square	*P*-value
How often do you commit to eating 3 main meals and 2 snacks?	5.119	0.275	8.756	0.068
How often do you eat fast food such as pizza, burgers or as daily main meals?	6.802	0.147	13.724	0.008^**^
How often do you eat French fries?	2.31	0.679	9.563	0.048^*^
How often do you eat snacks (such as popcorn, chips, so on)	9.977	0.041^*^	10.328	0.035^*^
How often do you eat fruits and vegetables?	2.719	0.606	2.429	0.657
How often do you eat healthy foods (eggs, whole wheat, vegetables and fruits, nuts,) in your daily meals?	10.076	0.039^*^	5.264	0.261
How often do you have 2-3 servings of milk or its products (such as laban, yoghurt, cheese)	6.849	0.144	14.931	0.005^**^
How often do you eat one or more serving units of eggs or meat per day?	7.639	0.106	9.248	0.055
How many spoons of sugar do you add to foods and drinks per day?	1.717	0.788	1.815	0.770
How often do you soft or sweetened beverages (juices, sodas, energy drinks, and so on)?	10.258	0.036^*^	8.284	0.082
How often do you eat pastries (cakes, donuts, bites, pastries, sweets, chocolate, so on)?	20.82	0.000^**^	17.737	0.001^**^
How often do you eat fast food because of boredom, distress and the desire to eat without hunger?	2.238	0.692	7.132	0.129
How often do you do 30 minutes of physical activity per week (such as walking, cycling, dancing, and so on.)?	11.814	0.019^*^	6.602	0.158
How often do you contribute in (cooking, laundry, or cleaning) at home?	58.246	0.000^**^	64.675	0.000^**^
How often do you contribute to daily activities such as (shopping grocery, neighborhood walk?	72.288	0.000^**^	32.707	0.000^**^
How many hours is your daily time sitting at work?	9.875	0.079	22.089	0.001^**^
How many times do you break while working during office hours (rest time 10 minutes)?	4.1	0.393	6.8	0.147
How many hours do you spend daily watching television or mobile phone for social media or playing video games?	0.832	0.842	1.658	0.646
How many hours do you sleep daily?	0.18	0.914	6.28	0.043^*^
Is there any change in your quality of sleep?	3.339	0.503	2.358	0.670
How many times do you feel stress or anxiety in a day?	5.909	0.206	18.334	0.001^**^
Do you smoke cigarettes, shisha or almuasal or electronic cigarettes?	21.473	0.000^**^	18.233	0.001^**^
Do you received any help to maintain a healthy lifestyle from family and friends?	4.078	0.396	8.339	0.080

**Figure 1:**
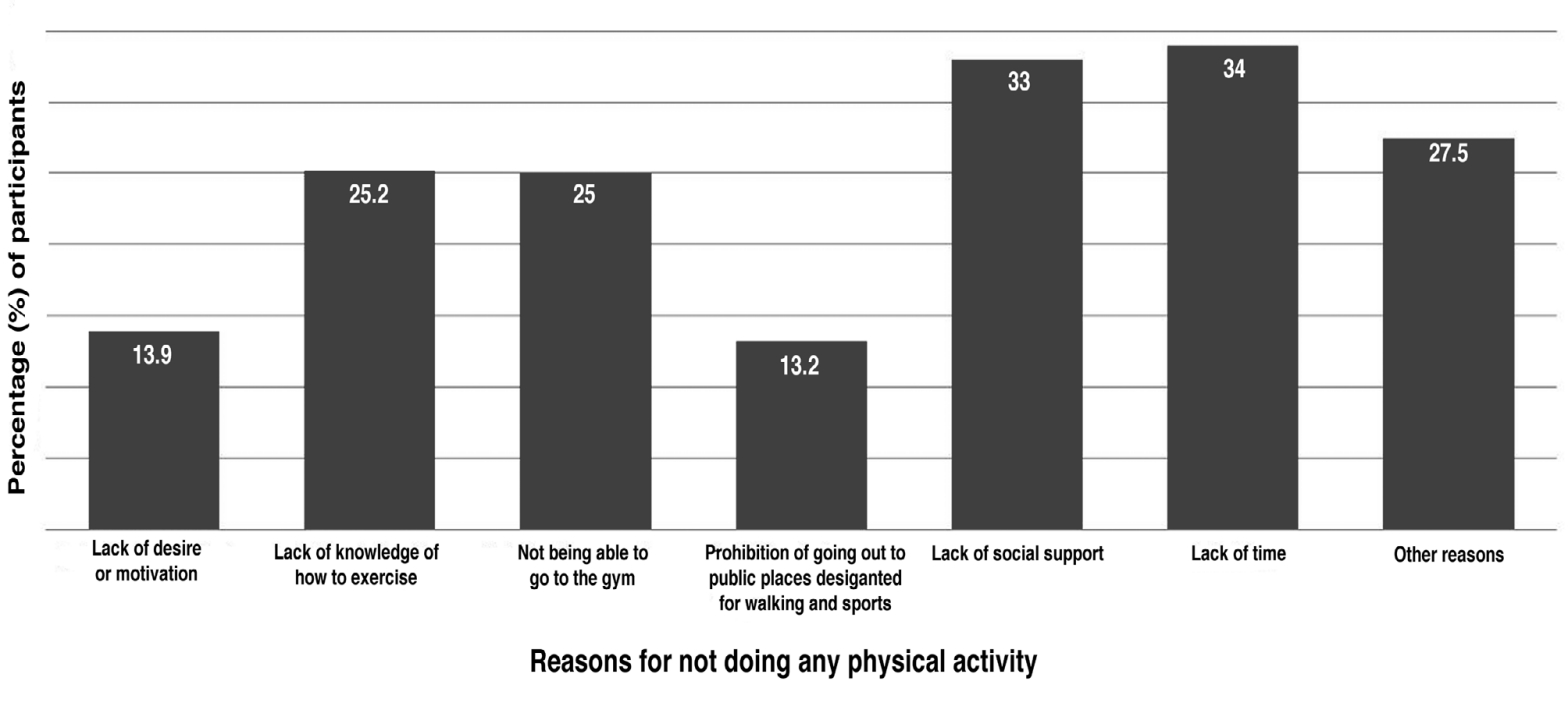
- Reasons to not doing any physical activity during quarantine (N=476).

Study participants reported many changes in their lifestyle specially the physical activity which decreased during the quarantine. Approximately 70% of them engaged in different physical activities at home using machines such as treadmills or bikes, or mobile sports applications. Approximately 30% of them did not engage in any physical activity. The lack of physical activity was attributed to several reasons such as an inability to go to the gym and the lack of knowledge on how to exercise (Figure 1).

## Discussion

Coronavirus disease 2019 pandemic has led to many alterations in the daily routines of individuals worldwide. Authorities in all countries, including Saudi Arabia have dealt with this pandemic to limit its spread. The Saudi authorities have implemented both strict infection control alongside partial or complete closures of sectors such as schools, universities, markets, and restaurants, forcing people to study and work from home.

During the quarantine, many study participants lost weight. This can be explained by their tendency to eat healthy foods so as to preserve their health and avoid infection with SARS-CoV-2. However, other studies showed that many people gained weight during lockdowns, which may be due to changes in their daily routines and lifestyles.^16^

The results of the current study showed many changes in eating habits, food shopping, consumption patterns and lifestyle behaviors. There was a significant increase in the daily consumption of snacks, foods and beverages high in sugar after quarantine compared to the quarantine period. And compared to studies carried out at the national level in Saudi Arabia as in the study of Al-Fawaz et al^17^ and Al-Kharashi^18^ who found that people changed their eating habits, increasing intake of snacks between meals and of sugary foods and beverages.

A study carried out in the United Arab Emirates, France, and Riyadh showed that there were changes in food consumption and purchasing habits, which worsened significantly during the COVID-19 quarantine. Changes in eating behaviors occurred due to anxiety, fear, or boredom and increased mood-driven eating.^18-20^ In comparison, the current study showed that the impact of quarantine due to the Corona virus had a negative impact on the shopping and food consumption patterns of the participants. This was reflected in the significant increase in the consumption of pastries and fast food. This may be due to boredom and distress.

The lifestyle of the study participants was negatively affected by the pandemic, affecting physical activity, and participation in activities such as shopping and neighborhood walks. Furthermore, study participants spent more time using social media and mobile phones, watching television, and playing video games during the pandemic than during the post-quarantine period. These results were consistent with previously published reports at national and global levels showing that lifestyle habits have been significantly and negatively changed due to the COVID-19 pandemic.^17-19^ Studies have demonstrated that individuals have significantly reduced the amount of physical activity and increased their daily time utilizing the internet, social media, and electronic gadgets. Quarantine has also resulted in increasing the time spent at home, which includes online education and remote work, limiting outdoor time and physical activity. Individuals have engaged in food stockpiling due to restrictions on grocery shopping, which has led to increased body weight and a negative impact on the public health.

The quarantine period altered the participants’ sleep hours and sleep quality compared to the post-quarantine period. Respondents reported decreased hours of sleep and increased sleep-wake disturbances, which may be caused by long working hours. Participants attributed the causes of anxiety and stress to feelings of malaise/boredom/loneliness as a result of quarantine, and concern on family, friends or themselves infected with COVID-19. This is consistent with findings from previously published reports from western and domestic countries that have shown that sleep quality and number of hours have been significantly altered during the pandemic.^18-20^

We investigated relationships between sociodemographic factors (age, gender, educational level, family allowance, marital status, and BMI) and health habits and behaviors. The results of the current study indicated that age had a significantly positive effect (*p*<0.05) on their health habits and behaviors during and after quarantine, with older people adopting healthier habits and behaviors. Body mass index also had a significant negative effect (*p*<0.05) on health habits and behaviors during and after the quarantine, with subjects with a higher BMI reporting lower health habits and behaviors. Compared with the results of other studies at the level of the United Kingdom, Spain, the United Arab Emirates and Italy, which showed a negative effect of high body mass index and a positive effect of age on healthy habits and behaviors.^2,19,22,23^

Therefore, we recommend encouraging people to reduce snacking and choose foods rich in vitamins by consuming more fruits, vegetables, nuts, and dairy products. Maintaining healthy lifestyle habits and behaviors, as well as improving the diet to increase the efficiency of the immune system and increasing physical activity during future lockdowns will have a beneficial impact on the quality of life of individuals worldwide.

### Study limitations

The data should be documented and investigated in larger population studies in the future, given that the quarantine led to the use of the 2010 Saudi census that prompted us to work on a small sample size. Although the questionnaire was disseminated via various social media (WhatsApp, Twitter, and via the university e-mail with the help of KAU’s Research Services Unit) to all male and female participants, few males answered the questionnaire, which in turn affected the number of the sample. This is in addition to publishing the questionnaire in various regions of the of Saudi Arabia, but the focus was on the city of Jeddah.

In conclusion, this study demonstrated the changes in the dietary and lifestyle habits of Saudi adults throughout and after COVID-19 quarantine, with some positive and some negative impacts, such as greater consumption of unhealthy foods among women. However, healthy lifestyle behaviors during COVID-19 quarantine increased significantly in women, with reduced intake of sweetened juices and soft beverages. This study also revealed the reasons for lifestyle changes during quarantine as the result of the COVID-19 pandemic. Participants’ failure to engage in physical activity during quarantine was owing to a lack of time due to long working hours, which in turn affected their sleep patterns, in addition to boredom and frustration during quarantine.
